# A case study of a strategic initiative in pediatric rehabilitation transition services: An insiders' perspective on team principles and practices

**DOI:** 10.3389/fresc.2022.999973

**Published:** 2022-12-09

**Authors:** Gillian King, Laura R. Bowman, C. J. Curran, Anna Oh, Laura Thompson, Carolyn McDougall, Dolly Menna-Dack, Laura Howson-Strong

**Affiliations:** ^1^ Bloorview Research Institute and Department of Occupational Science and Occupational Therapy, University of Toronto, Toronto, ON, Canada; ^2^ Holland Bloorview Kids Rehabilitation Hospital and Department of Occupational Science and Occupational Therapy, University of Toronto, Toronto, ON, Canada; ^3^ Holland Bloorview Kids Rehabilitation Hospital, Toronto, ON, Canada

**Keywords:** parallel principles, relational practice, co-design, solution-focused communication, humanism, teamwork, shared leadership

## Abstract

**Aims:**

The aim was to describe an innovative initiative that took place in a pediatric rehabilitation hospital. The goal of this organization-wide strategic initiative, called the Transition Strategy, was to improve service delivery to children/youth with disabilities and their families at times of life transition. The research question was: What are the key elements that have contributed to the success of the Strategy, from the perspective of team members? The objectives were to describe: (a) the guiding principles underlying team functioning and team practices, (b) key enablers of positive team functioning, (c) the nature of effective team practices, and (d) lessons learned.

**Methods:**

A holistic descriptive case study was conducted, utilizing historical documents, tracked outcome data, and the experiences and insights of multidisciplinary team members (the authors). Reflecting an insiders' perspective, the impressions of team members were key sources of data. The perspectives of team members were used to generate key teamwork principles, enablers of team functioning, team practices, and key learnings.

**Findings and Discussion:**

Team members identified four guiding humanistic principles (respect, support, partnership, and open communication). These principles underpinned three novel practices that contributed to team effectiveness in the eyes of team members: supportive relational practices, human-centered co-design, and solution-focused communication. Key enablers were the relational style of leadership, and a team climate of innovation, autonomy, and trust, supported by the organizational vision. This team climate fostered a sense of psychological safety, thereby encouraging both experimentation and learning from failure.

**Conclusions:**

This article provides information for other healthcare organizations interested in understanding the Strategy's value and its implementation. It provides a practical example of how to adopt a humanistic approach to health care, leading to both innovative service development and thriving among team members.

## Introduction

“*Fallen through the cracks*”, “*left stranded*”, and “*lost in the system*”: These metaphors describe the demoralizing state of affairs experienced by many youth with disabilities as they transition to adulthood (1–3). They also indicate the widespread need for an initiative or approach to address the shortcomings of the healthcare system, as it often fails to address the needs and aspirations of children and youth with disabilities, particularly at times of transition such as from post-secondary education to adult roles (4).

Transition is a complex and multifaceted process requiring partnerships among young people, their families, service providers, and healthcare organizations and systems (5). Since transitions are often challenging for youth with disabilities and their families, innovative organizational initiatives are needed to address issues arising during transitions to adult roles and adult healthcare systems. Accordingly, this article describes the principles and practices of an organization-wide strategy (the Transition Strategy), which was designed to provide evidence-informed services to support children and youth with disabilities at times of transition and to foster meaningful experiences and meaningful lives (6). In this descriptive case study, eight members of the Transition Strategy reflected on their experiences and, guided by Mathieu et al.'s model of factors influencing team effectiveness (7), identified the key team principles and practices that supported the Strategy's functioning, achievements, and outputs.

### The Transition Strategy

The context for this work was a Canadian pediatric rehabilitation hospital that is also an academic health science centre, meaning that it promotes the integration of research, clinical, and educational activities to achieve evidence-informed decision making and optimal client care (8). The Transition Strategy was a five-year, donor-funded initiative that aimed to explore, understand, and take action to promote participation and well-being for young people with disabilities. The Strategy adopted an innovative focus on the *process* of transition, addressing the psychosocial aspects of growing into adulthood through the design of youth and family interventions informed by a humanistic approach and life course perspective (6, 9, 10). Beyond the impact on children, youth, and families, the Strategy aimed to have an impact on the pediatric rehabilitation healthcare system by changing engrained practices.

The vision of the Strategy was to create a sustainable systems-wide model that “re-thinks rehabilitation” by moving from a deficit-oriented to strengths-based approach to transition programming (11–13). From this perspective, “successful transitions” refer to outcomes such as self-efficacy, self-determination, adaptation, and resiliency (14), rather than just a successful medical handover from pediatric to adult services. The Strategy's strategic goals were to improve existing transition services, and design new evidence-informed programs in partnership with children/youth, families, and community organizations, thereby building child, family, and organizational capacity. The objectives were to co-create a common Strategy vision and ensure every client has access to personalized services as well as a transition plan. Given the hospital's status as an academic health science centre, the Strategy had both research and clinical goals. These joint goals reflected an integrated knowledge translation strategy, where program design and delivery are research-informed, and research findings and recommendations are readily translated into clinical practice (8, 15).

Our thinking about the Transition Strategy was guided by complex adaptive systems theory (16), which has been applied to healthcare organizations and systems. Key features of complex adaptive systems include multiple intersecting parts, an evolving, self-organizing nature, and simple rules that encourage creativity and innovation (17, 18).

In the following section, we briefly review current literature on an organizational team model that specifies mechanisms of team functioning, shared leadership, and thriving at work. These key concepts emerged from and informed team members' discussions and reflections on their experiences in the Transition Strategy.

### Background literature on effective team functioning

#### Models of team functioning

There is a vast literature on effective team functioning and models of teamwork in various fields, including organizational management and health care. Our case study was guided by Mathieu et al.'s (7) model of organizational teams. This model views teams as dynamic, multilevel, complex systems, which aligns with our view of the Strategy as a complex adaptive system. Mathieu et al.'s review of the last decade of research on team effectiveness used an input-mechanism-output model, emphasizing compositional features of teams (i.e., the combination of members' characteristics), structural features (e.g., task scope and complexity, team interdependence), and mediating mechanisms.

Our interest was primarily in mediating mechanisms, defined as team members' affect, behavior, and cognitions (19). Mediating mechanisms include team processes, which refer to the interdependent activities that organize task work to achieve collective goals (7, 20). Other mediating mechanisms are considered to be emergent states, including team cohesion, trust, and team climate (e.g., innovation climate and psychological safety climate). Mathieu et al. (7) have called for more research on teams as fluid entities operating in dynamic situations, and for more research on emergent states.

#### Shared leadership

In Mathieu et al.'s (7) model, shared leadership is both a structural team feature and a mediating mechanism. Shared leadership is a dynamic and emergent phenomenon, where team members share leadership roles and influence (21). Meta-analyses have shown a positive relationship between shared leadership and team performance (21). Relational leadership is one form of shared leadership, referring to a leadership model emphasizing social processes of co-construction, through which team collaboration and change emerge (22). From this perspective, leadership is a relational practice involving co-creation and co-production (23, 24), where there is a focus on communication, caring, and thriving in the workplace. Relational leadership is considered to be essential for dealing with complex issues, such as the design of transition services, where sustainability is a desired outcome along with individual well-being, organizational flourishing, and social change (23).

#### Thriving at work

As discussed by Mathieu et al. (7), effective teams produce tangible outputs/products and provide team members with valuable experience and new learning (7), which can contribute to a sense of thriving in the workplace. “Thriving” refers to feeling energized, valued, and productive through dynamic connections with others in the workplace (25), and is associated with supportive coworker and leadership behavior, and perceived organizational support (26). As well, perceptions of trust, autonomy, meaning, and a positive work environment have been found to be associated with team performance, team members' confidence, work engagement, innovation, and the sustainability of an innovation (27). Positive workplace practices such as respect, support, and a sense of meaning are associated with a positive team climate and a climate of innovation (28). Thus, to summarize, thriving in the workplace is related to the relationships that exist among team members, as well as the support of leadership and a team climate of innovation.

### Article aim and objectives

Our research question was: From the perspective of team members, what are the key elements that have contributed to the success of the Strategy? The specific objectives were to describe: (a) guiding principles underlying team functioning and team practices, (b) key enablers of positive team functioning, (c) the nature of effective team practices, and (d) lessons learned. These teamwork principles, enablers, team practices, and lessons learned can provide direction for others on how to co-design a successful innovation and encourage a sense of thriving among team members.

## Methods

### Case study design

The case under study was the Transition Strategy. We adopted Yin's (29) definition of a case study as an investigation of a contemporary phenomenon within its real life context. Case studies are appropriate when there is an interest in understanding how a complex real-life phenomenon occurs (29–31), particularly when the phenomenon and important contextual variables are not well understood (32), which is the case for the Transition Strategy. We adopted a descriptive case study approach, which involves describing the case, the sources and methods of data collection, and the findings (29). Ethical approval was not required for this study, as it was seen as a quality assessment.

**Figure 1:**
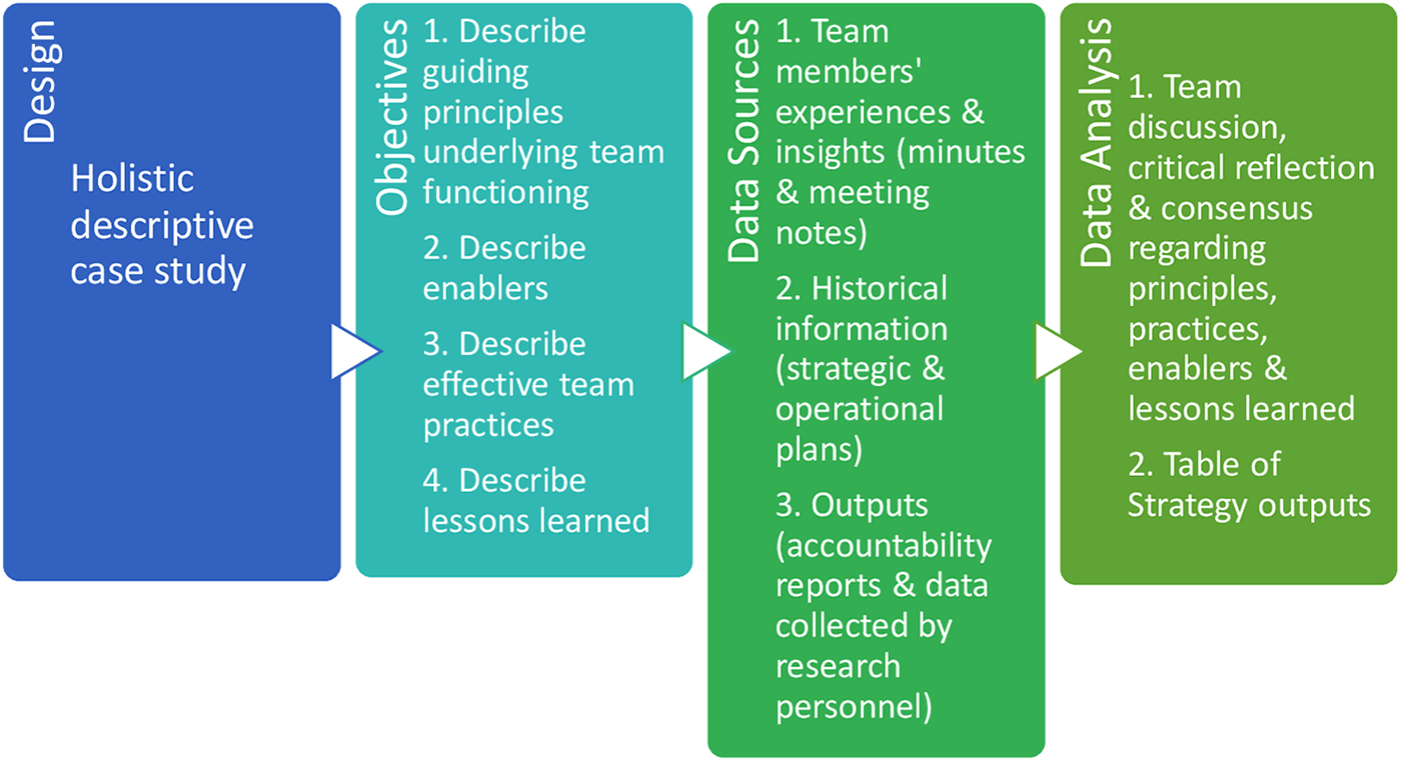
Case study protocol.

Case study protocols capture the study design, objectives, data sources, and data analysis procedures (29). As shown in our protocol (Figure 1), we took a holistic descriptive case study approach (29), using a single case to describe a unique phenomenon as a unit in a real-life context. As well, the case study was intrinsic—selected on its own merits, given the uniqueness of the Strategy (31). Similar to Di Pelino and colleagues (33), the perspectives of team members constituted the units of analysis (34), and the data consisted of their own direct first-hand experience in implementing the Strategy, as well as feedback they had received from other stakeholders and end users, including parents and youth who were receiving transition services.. Thus, the impressions of team members (the authors of this paper), who had clinical and research roles, were key sources of data (31). The perspective taken was that of the “insider”—an “emic” perspective that reflects an ethnographic approach (35).

### Data collection

As shown in the study protocol (Figure 1), the experiences and insights of team members were captured in minutes and meeting notes. Team members discussed team functioning, core practices, and lessons learned at retreats, monthly clinical-research team meetings, and at the end of project implementation, when the idea arose about writing an article to share our experiences and insights with others. Team members’ understanding of the implementation of the Transition Strategy was also informed by feedback they received from other clinicians implementing the Strategy, parents and youth receiving the services and engaged in advisory roles, and other organizational colleagues and upper management. This feedback came to the team from various discussions, through email, and from quality assurance surveys evaluating new programs.

Case studies routinely use multiple sources of data (29). As shown in Figure 1, although the experiences and insights of the team members were the primary sources of data, we also utilized historical documents (strategic planning documents, operational plans, meeting minutes, and other organizational documents) and tracked output information (from annual accountability reports, the organizational decision support system, and as collected by Strategy research personnel).

### Data analysis

The data analysis involved team discussion, critical reflection, and consensus regarding principles, practices, and enablers of the Strategy, as well as lessons learned. We began with a reflective stance, taking an inductive approach to our process. In addition, published literature on team functioning, including Mathieu's model of factors influencing team effectiveness (7), was also used to reflect on enablers and outcomes.

First, the perspectives of team members were used to generate key teamwork principles, enablers of team functioning, team practices, and key learnings. The first author, who is a researcher, developed the initial principles and practices based on key themes that arose repeatedly in discussions held at retreats, team meetings, and meetings held to draft this paper. Other authors wrote sections of text describing features and insights gained from the Strategy stream in which they were most involved. Consensus on key insights was achieved through discussion and reflection over monthly meetings held over a 6-month period, as the article was drafted. As the team generated ideas about thriving and relational leadership, research team members brought relevant literature to the full team for discussion. There were multiple check-ins, as team members read drafts of the article, to ensure the written representation fit their understanding of events and experiences.

Thus, team members critically reflected on their experiences over a period of prolonged engagement, which contributes to the trustworthiness of case study research (30). Triangulation among team and the use of multiple sources of data also serve to enhance credibility (36). Team members can be considered key informants, as they had been involved with the Strategy since its inception, and were able to report on clinical, client/family, and organizational perspectives.

### Case description

Here we consider the structure and outputs of the Transition Strategy. It is important to provide evidence of successful clinical and research outputs to justify the assertion that the Strategy was indeed an effective innovation.

The Transition Strategy was overseen by a steering committee co-led by a director and a family leader (a parent of a child with a disability). This committee included Strategy team members, family leaders and youth leaders, executive sponsors, community partners from the adult sector, and physicians. Team members were seconded from other roles in the organization based on their expertise, attitudes, drive, and passion, as well as their ability to build community partnerships, collaborate in interprofessional teams, and function as systems-level change agents.

An initial planning retreat led to the formation of five strategy streams for the development of evidence-informed services and the co-discovery of knowledge: Bridging to Adulthood (which builds innovative services and strong community partnerships to support youth transitioning to adult roles and settings), Employment Participation (services designed to promote participation in a range of typical early employment experiences), Starting Early (providing services, information sharing, and connections to support families and their young children in their first and early life transitions), Youth Engagement (providing leadership development and paid employment opportunities for youth with disabilities), and Solution-focused Coaching (a cross-cutting stream informing all the others). A co-design approach, reflecting relational leadership (22), was taken to identify this set of service streams.

**Figure 2:**
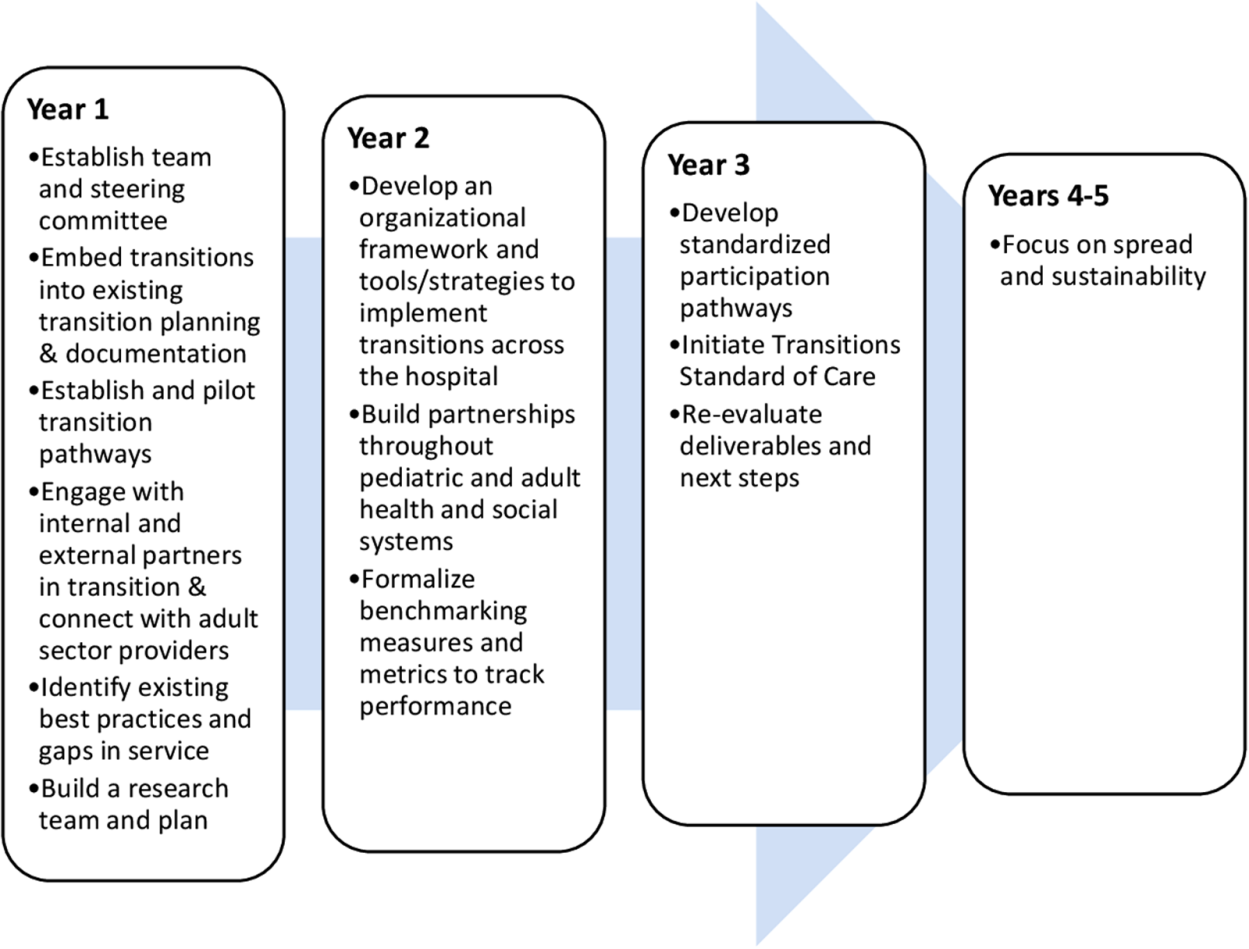
Yearly goals for the Transition Strategy.

The Strategy's annual goals are presented in Figure 2. These goals built upon the knowledge, connections, and practices developed in the preceding years to establish, implement, embed, and then sustain the learning achieved over time. The first year focused on establishing and grounding the Strategy to set the Strategy streams up for success. Years two and three focused on the development of new services built on transition best practices, along with the standardization of tools and procedures to facilitate transitions and evaluate the Strategy's success and impact. The final two years focused on spreading the established practices across and beyond the organization and finding ways to sustain these changes by embedding them in existing systems or proposing new teams, processes, or systems.

Outputs from the Strategy's activities are summarized in Table 1. Partnership/ collaboration activities led to the development of 32 new partnerships with external organizations, along with 23 new capacity building efforts developed in partnership with these organizations. More than 2,325 clients received clinical services, and 18 new or expanded programs were offered, along with 17 types of transition pop-up events (described later). Over 1,800 staff (internal and external) received training in solution-focused coaching and principles of solution-focused communication, and 23 student placements were offered, in addition to workshops and interprofessional education sessions. To date, research and program evaluation outputs included 49 conference presentations and 9 peer-reviewed journal publications. For example, a recent publication concerned pathways to employment for youth with disabilities (37).

**Table 1:** Summary of key outputs from activities of the Transitions Strategy^a^*.*

Activity Area	Outputs
Partnership/Collaboration	32 new partnerships, 23 capacity building efforts
Clinical Services	>2,325 unique clients served, 17 unique transition pop-up clinics offered, 8 expanded programs, 10 new programs, 3 new youth facilitator positions
Education/Training	883 internal staff and 926 individuals from partnering institutions trained in solution-focused coaching and communication 23 academic student placements, 27 unique workshops offered, 11 interprofessional education sessions delivered
Research/Program Evaluation	49 conference presentations accepted, 9 peer-reviewed publications

^a^ from the start of the initiative to October 2022.

## Findings and discussion

### Objective 1: Guiding principles underlying team functioning and team practices

**Figure 3:**
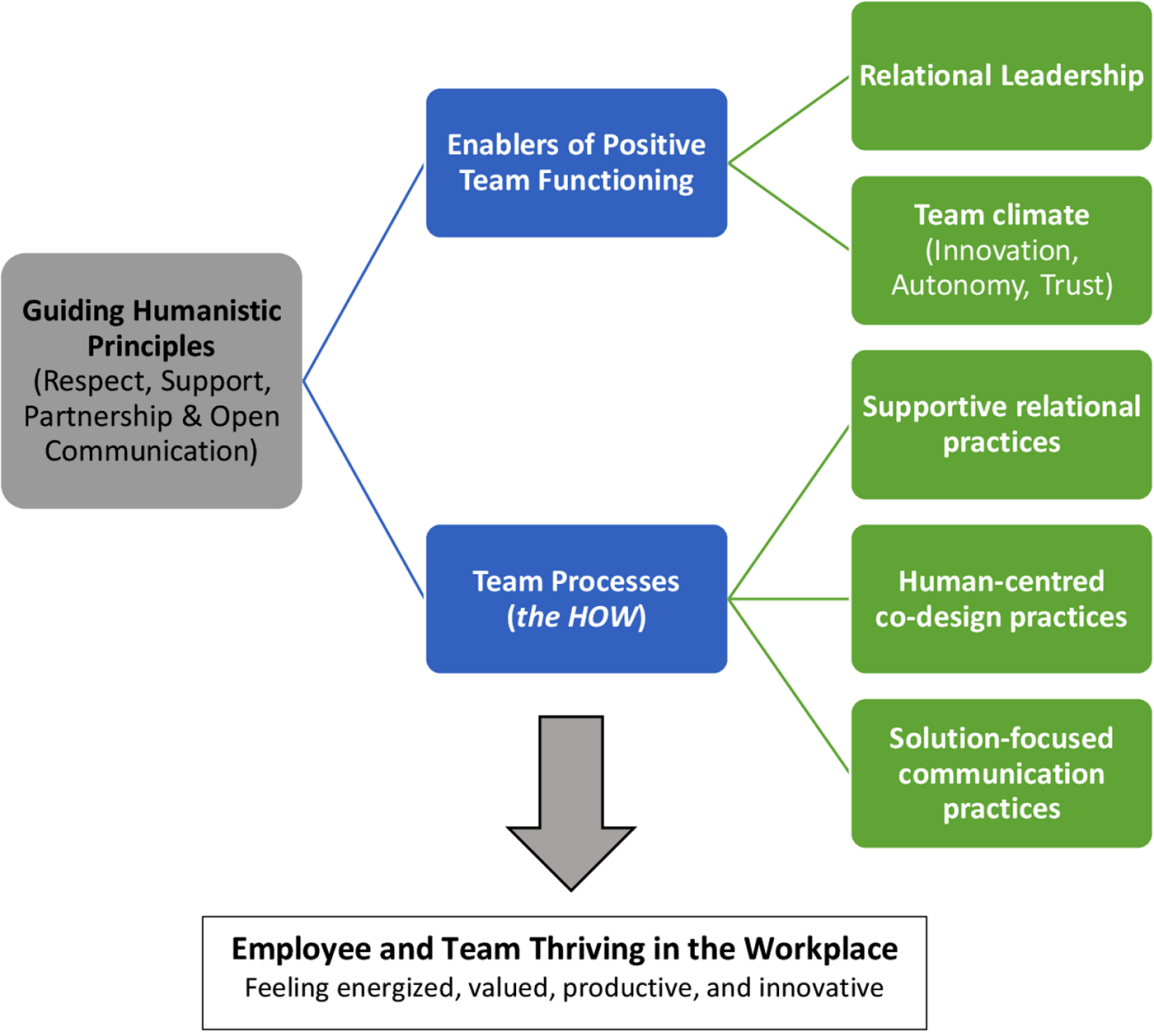
Guiding principles underlying team functioning and team practices in the Transition Strategy.

Figure 3 visually represents the Strategy's principles, key enablers of positive team functioning, and the team practices considered crucial to team functioning. As shown in this figure, the guiding humanistic principles were respect, support, partnership, and open communication. Team members valued genuine partnerships and collaboration, as well as an environment characterized by trust and respect. These humanistic values align with humanocracy theory, which highlights the importance of human values and principles in the workplace (e.g., the satisfaction of human needs through respect, and having control, support, and the opportunity to engage in challenging work in a safe environment) (38). Humanocracy theory emphasizes that management can be people-centered (38), reflecting the widely adopted philosophy of client/family-centered care in pediatric rehabilitation (39). This correspondence between how one is treated by colleagues and leadership, and how one works with others clinically, has been called “parallel processes” (40).

Parallel processes refer to features that are common to effective relationships both among managers and staff, and between service providers and clients (40). The common features of all forms of effective relationships include engagement through listening and attending, good communication, empowerment, and strength-building (40). Thus, both client-practitioner and practitioner-organization relationships play a fundamental role in supporting effective service provision (11). The idea of parallel organizational management and clinical processes serves to connect the ideas of team thriving and transformation of services: teams thrive when humanistic principles are adopted, and their thriving contributes to the development of innovative services and offerings.

### Objective 2: Enablers of positive team functioning

The team identified two main enablers of positive team functioning, with the first being the relational leadership style of the Strategy director. The director displayed a shared leadership approach, in which leadership roles are shared both formally assigned and informally adopted and encouraged (41). Kurucz et al. (42) has described leadership as a process of social engagement and the formal leader as someone who helps others collectively redefine what is seen as valuable, in light of various challenges. Leader inclusiveness promotes psychological safety and sets the stage for organizational learning (43).

Second, a team climate of innovation, autonomy, and trust, reflecting the broader organizational culture, was viewed as a key enabler of the Strategy's success, as it allowed team members to experience a sense of psychological safety. High quality relationships in the workplace foster psychological safety, which, in turn, is related to learning behaviors in organizations (44). Having shared goals, shared knowledge, and mutual respect foster psychological safety and enable team members to engage in learning from failure (45). Seeing failure as a chance to learn reflects the Strategy's humanistic and strengths-based perspective, and the effective practices adopted by the team.

### Objective 3: Nature of effective team practices

Here we consider supportive relational practices, human-centered co-design, and solution-focused communication, as shown in Figure 3. Each of these practices reflects a relational leadership model (23) and a team climate characterized by innovation and psychological safety (44). Table 2 provides examples of Transition Strategy models and programs that illustrate each of these practices, along with evidence of their impact.

**Table 2:** Illustrations of effective team practices.

Effective Team Practices	Illustrative Service Delivery Models and Programs	Impact
Supportive relational practices	Transition Strategy team members were excited about the Project SEARCH model's potential to transform employment programming for transition-aged youth. They introduced Project SEARCH to senior leadership and were enthusiastically supported to explore the transition-to-work model with potential partners, including the local school board, community developmental service organizations, and other internal partners. Leadership support included networking to promote partnership development, and leveraging short term funding.	Project SEARCH has become part of the hospital's strategic priorities concerning inclusive employment in the workplace. The hospital has also taken a leadership role in the provincial coordination of Project SEARCH site development for Ontario, Canada.
Human-centred co-design practices	Staff members used the human-centred co-design process to create a new “Transition Pop-Ups” service delivery model. After some trial-and-error experiences, what emerged was an innovative Transition Pop-Up service delivery model that offers comprehensive sets of resources, and through which the hospital tracks the demand for specific transitions-related needs from clients, families, and community partners. In this model, community partners, lived experience mentors, and internal staff with relevant knowledge and experience co-facilitate experientially-based sessions that address identified topics of interest. These sessions involve the following core elements: (a) community partnerships (authentic relationships, joint accountability), (b) information sharing, (c) peer mentorship, (d) action (on-the-spot completion of a key transition task), and (e) warm handovers to adult agencies (before pediatric graduation).	The Transition Pop-Up service delivery model has been systematized and recognized as a Leading Practice by Accreditation Canada.
Solution-focused (SF) communication practices	One example of embedding SF communication practices in transition programming involved the Starting Early stream, which offered workshops for parents/caregivers of children aged 7 and under. These workshops focused on transition-related skill building within family environments and providing connections with parents/caregivers with lived experience. The workshop series included a session on SF communication for parents, which provided opportunities for them to engage in SF activities and conversations. This session was co-designed by staff, parents with lived experience, and past participants.	An evaluation survey of the workshop series found notable increases in participants’ confidence in using a SF approach when encountering challenges. One parent noted that the SF communication session had a “transformative impact” on her family's interactions with medical teams, as conversations with service providers had shifted to focus on strengths and solutions, vs. problems.
		

#### Supportive relational practices

The hospital has a culture of innovation, which is exemplified and promoted by the senior leadership. Ideas are welcomed, space and time are allowed for the exploration of ideas, and guidance is provided at key moments. In this regard, members of the Strategy experienced many challenges together, such as developing programs that were considered to be unfeasible by the steering committee and other key stakeholders. Some of these instances were viewed, in hindsight, as positive learning experiences.

Team members felt able to engage in lively debate on various issues. Despite opposing views, there was a team atmosphere of respect and a safe working environment in which team members could actively engage through “transparent and respectful conversation”. Free exploration of ideas promoted ongoing innovation. For example, prompted by the COVID-19 pandemic, virtual programming was adopted but, as the pandemic wore on, it became apparent that families felt “virtual fatigue”. Team members adjusted their approach by offering hybrid services (a combined virtual and face-to-face approach), offering workshops on an individual basis, embedding workshop knowledge in other hospital initiatives (e.g., parent support networks, webinars), and sharing content through YouTube videos for families. This example illustrates the ability of team members to identify issues, brainstorm, and innovate in a supportive team environment, and to quickly move from what does not work to better solutions.

Another example, dealing with Project SEARCH, is presented in Table 2. Project SEARCH is an example of an innovative program aligned with the team's mandate. This project is an internationally adopted “best practice” transition-to-work model for students with developmental disabilities, which provides supported work experiences, life skills training, and employment planning (46). Inter-agency collaboration is a key ingredient of the model (47). Annually, up to 75% of Project SEARCH graduates transition to employment (46, 48).

#### Human-centered co-design practices

Human-Centered Design (HCD) refers to principles and methods that aim to create innovative solutions tailored to the needs and preferences of the end user (49). HCD has been employed in business organizations to collaboratively solve complex problems and drive service innovation, and has been implemented in a variety of healthcare settings, including disease management and health education (50). To date, HCD has been used to co-design transition support services concerning hospital discharge/transfer of care and to create standardized products/tools (51, 52), but not to design innovative procedures or strategies.

The team embraced HCD principles from IDEO.org (“design thinking”) as a foundation for program development and refinement, as these principles reflected the team's humanistic perspective and relational leadership approach. They used a five-stage HCD process developed by the Stanford d.school (a design thinking institute based at Stanford University): (1) empathizing with and understanding the needs of the users (empathy); (2) constructing a point of view based on the needs and insights of the users (define); (3) brainstorming ideas and generating creative solutions (idea); (4) building a physical representation of the ideas (prototype); and (5) testing ideas for feedback and iteration (test).

Over 100 individuals participated in the co-design process, including stakeholders, service providers, adult sector partners, and youth and families with lived experience. The following HCD principles were adopted: (a) assuming a beginner's mindset (a stance of curiosity), (b) approaching interactions with empathy, (c) co-creating ideas and prototypes (i.e., valuing lived experience and adopting a participatory stance), and (d) engaging in an iterative process, based on recognition that plans need to be flexible with respect to implementation (49). Co-creation is non-linear, iterative, and exciting; it is associated with the belief that solutions are emergent, and that it is alright to “not know”. One specific example, dealing with the creation of a new “Transition Pop-Ups” service delivery model, is presented in Table 2.

#### Solution-focused (SF) communication practices

SF communication refers to a set of practices based on humanistic principles, where listening and communication are considered central to client-provider and team member relationships, client/family-centered care (39), and effective service delivery (53–55). As a result of the Strategy, training in SF communication took place across the hospital. This training facilitated knowledge and confidence in using SF coaching techniques, and provided a common framework for collaborative solution-finding among teams across the organization (56, 57). A research study indicated that the training led to greater use of strengths-based language in clinical documentation, strengths-based initial assessments and intake interviews, and strengths-based activities in programs for both youth and family members (56, 57). Table 2 provides an example of the use of SF communication in the design of workshops in the Starting Early stream.

### Objective 4: Lessons learned

Here we discuss two key learnings that reflect the Strategy's humanistic principles and practices. The first was recognizing the value of embracing the inherent messiness of the process involved in designing new services, as well as the inherent messiness of transitions themselves. Embracing this messiness and uncertainty was seen as a key ingredient for healthy and “real” transitions, which, by their very nature, are stressful and highly individual. Given the widespread use of transition guides and skills checklists that are intended to make things “easier” for the healthcare system, it can be argued that the system paradoxically oversimplifies and overcomplicates transitions by not specifically adopting a client-centered perspective, even though this is often espoused. A client-centered perspective requires understanding the highly personal nature of a given youth's transition needs, resources, and supports. The Strategy team struck a balance between systematizing (for equity) and individualizing (for client-centeredness and humanism) by recognizing the real-world messiness of transitions and by listening to clients mindfully and authentically (58), in ways that validate their experiences and foster hope and resiliency.

The second key learning was the value of the Strategy for the organization, clients and families, and team members themselves. On an organizational level, the complement of clinical programs increased due to the efforts of the Strategy. New initiatives were developed in partnership with other service organizations or were adopted in other parts of the hospital, indicating the integration and sustainability of the offered services. One noteworthy example of sustainability and spread is the Solution-focused Coaching stream, which is now embedded hospital-wide and is part of the foundational training for all new staff members. Another example is Project SEARCH, which launched as a transition initiative but is now recognized as a key element of the organization's human resources function.

The value of the Strategy for clients/families can be seen in the “best practice” of supporting families from their first contact with the hospital and throughout their journey to adult systems and adult roles. Transition-related skills are seen as life-long capacities that develop over time within the family and real-world environment (6). Trained family leaders are now available at the hospital to provide information and support regarding strategies, resources, and connections to peer mentors and community agencies, in order to ease the transition journey.

Last, the value for team members was demonstrated by their commitment and ongoing engagement. They had rich learning experiences contributing to their personal and professional growth and sense of thriving in the workplace. By being open to the needs and hopes of clients/families, team members felt they were guided and led by families in essential ways, reflecting the notion of client/family-centered care. Through the integrated knowledge translation approach adopted in the Strategy, team members came to value research more highly and were in an ideal situation to integrate findings into practice in immediate ways.

## Overall discussion

This article adopted an insider perspective to conduct a case study of the Transition Strategy, a five-year donor funded initiative that viewed transitions from a strength-oriented, systems perspective. The aim was to uncover and describe the key elements contributing to the success of the Strategy in the eyes of team members, who were the authors. Guiding principles underlying the functioning of the team were considered to be respect, support, partnership, and open communication, which align with principles identified in the literature on client/family-centered care (39). Key enablers were the Director's relational leadership style and the team climate, which contributed to a sense of psychological safety, trust, and thriving in the workplace. Several innovative team practices were identified, including supportive relational practices, human-centered co-design, and solution-focused communication. These practices reflect standardized processes generated by the team to enhance their effectiveness. Two key lessons learned were the inherent messiness of the transition process, which therefore requires an individualized approach, and the value of the Strategy for the many stakeholder groups involved with the initiative. Thus, this article provided an in-depth description of the variables that, in the eyes of team members, were responsible for team outputs and service transformation (attesting to the effectiveness of the Strategy), and members' sense of thriving in the workplace.

### Limitations

This article has several limitations. Foremost, aside from personal anecdotes, we do not know the wider impact of the Strategy on clients and families. We did not conduct formal interviews with key informants; rather, we adopted an “insider” perspective where key informants were authors who collaboratively contributed their experiences and insights to this paper. Nonetheless, this article provides other organizations with a vision, starting point (key principles, enablers, and practices), and initial evidence for the utility of an organization-wide strategy with a focus on transforming services for young people with disabilities and their families, which has had the concomitant benefit of facilitating team members' sense of thriving in the workplace. There are important teamwork and leadership implications for others interested in implementing a similar initiative in health care, whether it be about transitions or another important gap concerning needs or issues in service delivery.

### Implications

This article has implications for both public and private healthcare organizations. The principles, enablers, team practices, and lessons learned can be informative for others seeking to develop a strategic initiative that relies on the effective functioning of a team. In addition, interested organizations can use the Strategy's operational plans for guidance in how to set milestones for an organizational initiative. The annual operational goals were to *establish* the streams of the Strategy, to *implement and evaluate* new services and standardize tools and procedures, to *spread established practices* within the organization and beyond, and to *sustain* these changes by embedding them in existing or newly created processes or systems. This general process of *establish, implement and evaluate, embed*, and then *sustain* can be used by other organizations to develop milestones for similar initiatives that aim to improve service design and delivery on a system-wide level.

From a leadership perspective, one key take-away message is that a coordinated and directed initiative, operating in accordance with a humanistic philosophy, can develop innovative programs and facilitate learning among team members. Health care can be potentially humanized through strategic initiatives that build strong, flexible, interprofessional teams involving multiple stakeholders and partners. A team that thrives interpersonally, psychosocially, and practically can provide an ideal environment for the design of services that provide optimal client care. It should also be noted that the Strategy took place in a relatively ideal organizational situation, with ample funding and positive, ongoing relationships with partnering organizations. From the start, team members had many practical foundations in place, including expertise and established working relationships. As well, they were receptive to a relational model of leadership, and had a strong commitment to the Strategy. Thus, the present findings may not directly translate into other publicly funded organizational environments.

## Conclusion

This case study of a workplace team brought together to design, deliver, and evaluate a suite of evidence-informed, transitions-oriented services has shown the value of humanistic principles and relational leadership, as well as supportive relational practices, human-centered co-design practices, and solution-focused communication practices. Our hope is that others can learn from and be inspired by the guiding principles and team practices that embodied the learning experiences of team members and contributed to their sense of thriving in the workplace.

## Data Availability

The original contributions presented in the study are included in the article/supplementary materials. Further inquiries can be directed to the corresponding author/s.
